# Norovirus GII.4 Strains and Outbreaks, Australia

**DOI:** 10.3201/eid1307.060999

**Published:** 2007-07

**Authors:** Elise T.-V. Tu, Thanh Nguyen, Phoebe Lee, Rowena A. Bull, Jennie Musto, Grant Hansman, Peter A. White, William D. Rawlinson, Christopher J. McIver

**Affiliations:** * University of New South Wales, Sydney, New South Wales, Australia; † Prince of Wales Hospital, Sydney, New South Wales, Australia; ‡ New South Wales Department of Health, Sydney, New South Wales, Australia; § National Institute of Infectious Diseases, Tokyo, Japan

**Keywords:** Norovirus, outbreaks, gastroenteritis, Australia, letter

**To the Editor:** Viral gastroenteritis affects millions of people of all ages worldwide, and some seasonality has been observed in outbreak occurrences (*1*–*3*). During early 2006 in New South Wales (NSW), a marked increase in outbreaks of gastroenteritis occurred (Figure): 155 outbreaks were reported during the first 5 months compared with 88 outbreaks during 2005. During the first 5 months of 2006, the Enteric Pathogens Laboratory–South Eastern Area Laboratory Services (EPL-SEALS) recorded an increase in norovirus in stool samples, detected by using an enzyme immunoassay (IDEIA Norovirus, DakoCytomation, Cambridgeshire, UK). From January through May 2006, the proportion of samples positive for norovirus increased successively: 0/47 (0%), 1/73 (1.4%), 5/169 (3.0%), 8/106 (7.5%), and 93/413 (22.5%). This trend followed the increasing reports of outbreaks made to the NSW Department of Health (Figure). In May, the rate of norovirus detection (22.5%) was significantly greater than that of any other pathogen (Fisher exact test, p<0.0001), including intestinal parasites, foodborne bacterial pathogens (*Salmonella,* *Shigella,* and *Camplylobacter*), and enteric viruses (rotavirus, adenovirus, and astrovirus).

**Figure:**
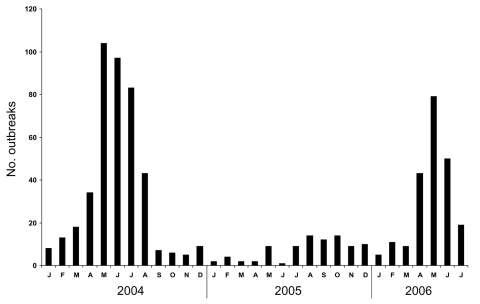
Number of outbreaks reported to the New South Wales Department of Health, January 2004–July 2006.

In April 2006, the NSW Department of Health Public Health Real-time Emergency Department Surveillance System (PHREDSS) detected a significant increase in visits for gastroenteritis. This system records cases in real time for each visit to an emergency department from patient demographic information and syndromes diagnosed according to the International Classification of Diseases, version 9, Clinical Modification (ICD-9-CM) (*4*). Information collected came from a population of >4 million persons, predominantly in the Greater Sydney metropolitan region. During April and May 2006, >8,000 visits for vomiting and diarrhea were recorded in PHREDSS, which is ≈3,000 above the average number of cases for this period for the previous 5 years. During this 8-week period, reports of clinically diagnosed outbreaks in institutional settings also increased; 129 outbreaks affected >3,485 persons. This number of outbreaks is the highest ever reported in the April–May (autumn) period for this region since data for outbreaks (mainly from aged-care facilities, hospitals, childcare centers, and schools) were collected by NSW Department of Health in 2004. Furthermore, laboratories detected norovirus in 37 (28.7%) of 129 outbreaks investigated by public health authorities.

The stool samples examined by EPL-SEALS in May 2006 were from patients treated in public hospitals and nursing homes within the Eastern Sydney and Illawarra regions as well as referred samples from private laboratories serving the Greater Sydney region. Sequencing of a random selection of 15 positive samples representative of the diverse location of case-patients indicated that 10 (66.7%) of 15 were GII.4 genotype. The nucleotide sequence of 266 bp of the N terminus of the capsid gene closely resembled (98%) the sequence of Farmington Hills virus and was 100% identical over the same region to norovirus Rhyl440. The remaining positive strains belonged to GII.3 (4 of 15) and GII.12 (1 of 15). The latter strain, designated Schwerin virus, was previously isolated in outbreaks in Germany during 2000. Two of these sequenced samples were from nursing home outbreaks and typed as GII.3 and GII.4. The association with outbreaks of the remaining 13 sequenced samples is uncertain.

Norovirus epidemics occurred throughout the world in 2002 (*5*) and 2004 (*6*) and were characterized by the large number of persons affected, multiple routes of transmission, and persistence of endemicity despite rigorous control efforts. In Australia from 1997 to 2000, a period of high activity occurred, dominated by the GII.4 epidemic strain designated U.S. 95/96 (*7*). Farmington Hills virus was responsible for subsequent outbreaks in Sydney in 2002 and followed a decline in norovirus outbreaks during 2001. After the 2002 outbreaks in Australia, a period of low norovirus activity persisted before the 2004 emergence of another GII.4 variant, designated Hunter virus (*3*), which predominated in outbreaks in nursing homes and hospitals. More than 400 outbreaks affecting >15,000 persons occurred in NSW during 2004 (*8*). The Hunter virus was subsequently determined to be the etiologic agent in hundreds of outbreaks occurring in Holland (*9*), New Zealand (Gail Greening, pers. comm.), Taiwan, and Japan.

The occurrence of norovirus epidemics in the Australian autumn (March–May) contradicts the perception that the disease is strongly associated with the winter season, when the incidence of respiratory infections increases (*2*). Indeed, other norovirus outbreaks have previously been reported in the summer season (*1*,*3*).The alternating trend of high and low incidence of outbreaks (Figure) may be related to several factors, some of which may have been implicated in these outbreaks. Such factors include development of herd immunity to the dominating strain, which is short-term; emergence of an epidemic strain with no herd immunity; increased genomic variation due to point mutation or recombination (*10*); or other mechanisms.

## References

[1] Lopman BA, Reacher M, Gallimore C, Adak GK, Gray JJ, Brown DWG. A summertime peak of “winter vomiting disease.” Surveillance of norovirus in England and Wales, 1995 to 2002. BMC Public Health. 2003;3:13. 10.1186/1471-2458-3-13 12659651 PMC153520

[2] Mounts AW, Ando T, Koopmans M, Bresee JS, Noel J, Glass RI. Cold weather seasonality of gastroenteritis associated with Norwalk-like viruses. J Infect Dis. 2000;181:S284–7. 10.1086/315586 10804139

[3] Miyoshi T, Uchino K, Matsuo M, Ikeda Y, Yoshida H, Sibata H, Characteristics of norovirus outbreaks during a non-epidemic season. Jpn J Infect Dis. 2006;59:140–1. 16632921

[4] Muscatello DJ, Churches T, Kaldor J, Zheng W, Chiu C, Correll P, An automated, broad-based, near real-time public health surveillance system using presentations to hospital emergency departments in New South Wales, Australia. BMC Public Health. 2005;5:141. 10.1186/1471-2458-5-141 16372902 PMC1361771

[5] Widdowson M-A, Cramer EH, Hadley L, Bresee JS, Beard RS, Bulens SN, Outbreaks of acute gastroenteritis on cruise ships and on land: identification of a predominant circulating strain of norovirus—United States, 2002. J Infect Dis. 2004;190:27–36. 10.1086/420888 15195240

[6] Bull RA, Tu ET, McIver CJ, Rawlinson WD, White PA. Emergence of a new norovirus genotype II.4 variant associated with global outbreaks of gastroenteritis. J Clin Microbiol. 2006;44:327–33. 10.1128/JCM.44.2.327-333.2006 16455879 PMC1392656

[7] White PA, Hansman GS, Li A, Dable J, Isaacs M, Ferson M, Norwalk-like virus 95/96-US strain is a major cause gastroenteritis outbreaks in Australia. J Med Virol. 2002;68:113–8. 10.1002/jmv.10177 12210438

[8] Telfer B, Munnoch S. OzFoodnet—enhancing foodborne disease surveillance across Australia. In: Annual report. Sydney (Australia): New South Wales and Hunter Area Health Service; 2005.

[9] Kroneman A, Vennema H, van Duijnhoven Y, Duizer E, Koopmans M. High number of norovirus outbreaks associated with GGII.4 variant in The Netherlands and elsewhere: does this herald a worldwide increase? Euro Surveill. 2004;8.

[10] Bull RA, Hansman GS, Clancy LE, Tanaka MM, Rawlinson WD, White PA. Norovirus recombination in ORF1/ORF2 overlap. Emerg Infect Dis. 2005;11:1079–85. 16022784 10.3201/eid1107.041273 PMC3371806

